# Minimally Invasive vs Conventional Coronary Bypass Surgery for Multivessel Coronary Disease

**DOI:** 10.1016/j.atssr.2024.10.024

**Published:** 2024-11-14

**Authors:** Yichen Gong, Tong Ding, Xiaoxiao Wang, Zhongqi Cui, Hong Zhao, Song Wu, Yuanhao Fu, Hang Yang, Yunpeng Ling

**Affiliations:** 1 Department of Cardiac Surgery, Peking University Third Hospital, Peking University Health Science Center, Beijing, China; 2 Research Center of Clinical Epidemiology, Peking University Third Hospital, Peking University Health Science Center, Beijing, China

## Abstract

**Background:**

Despite sternum sparing and without cardiopulmonary bypass, the actual value of minimally invasive coronary surgery (MICS) is still debatable. This study aimed to compare the completeness of revascularization and intermediate-term outcomes of MICS with conventional sternotomy coronary artery bypass grafting (CABG).

**Methods:**

Two groups of 244 patients each receiving MICS-CABG and sternotomy-CABG between November 2015 and March 2019 were matched by propensity score matching. The completeness of revascularization and major adverse cardiovascular and cerebrovascular events (MACCE; a composite of death, myocardial infarction, stroke, or repeated target vessel revascularization) were compared between the groups.

**Results:**

In the MICS-CABG group, the percentages of bypassed vessels 2, 3, and ≥4 were 53.7%, 36.1%, and 10.2%, respectively. Completeness of revascularization (95.5% vs 96.3%; *P* = .65) was comparable between MICS-CABG and sternotomy-CABG groups. Postprocedural angiography revealed an overall patency of 96.2% (578/601) for the MICS-CABG group. At 5 years, rates of MACCE (19.9% vs 22.1%; hazard ratio [HR], 0.80; 95% CI, 0.49-1.32; *P* = .39), death (10.6% vs 12.9%; HR, 0.87; 95% CI, 0.46-1.65; *P* = .67), myocardial infarction (5.6% vs 4.2%; HR, 0.82; 95% CI, 0.27-2.52; *P* = .73), stroke (6.7% vs 6.6%; HR, 1.11; 95% CI, 0.43-2.86; *P* = .83), and repeated target vessel revascularization (1.9% vs 1.8%; HR, 0.85; 95% CI, 0.17-3.15; *P* = .84) were similar between MICS-CABG and sternotomy-CABG.

**Conclusions:**

MICS-CABG, which appeared to yield noninferior completeness of revascularization and intermediate-term MACCE compared with sternotomy-CABG, could be an alternative for patients with multivessel coronary diseases.

## Visual Abstract

**Figure:**
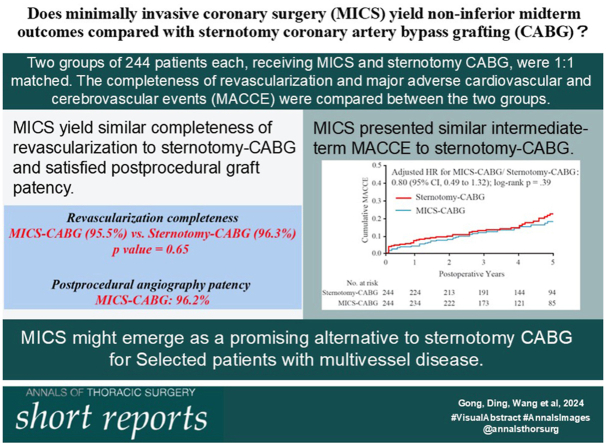

**In Short**

▪ The study indicated that minimally invasive coronary surgery yielded similar revascularization completeness and intermediate-term major adverse cardiac and cerebrovascular events.
▪ Minimally invasive coronary surgery might emerge as a promising alternative to sternotomy coronary artery bypass grafting for treatment of multivessel disease.

Although conventional coronary artery bypass grafting (CABG) remains the “gold standard” for treatment of multivessel disease,^1^ its disadvantages, such as potential sternum complications and more transfusion, are widely debated within the revascularization community.^2^

Minimally invasive coronary surgery (MICS), bypassing multiple conduits and graft configurations to various myocardial territories, preserved the applicability and durability of coronary bypass surgery while being sternum sparing and without cardiopulmonary bypass.^3^ During the last decade, many studies indicated that MICS-CABG is associated with improved postoperative outcomes, such as reduced infections, drainage, and transfusions.^4^ The MIST trial (NCT03447938), which was conducted by Ruel and colleagues with 88 participants per arm to investigate the early functional status of MICS-CABG using the 36-Item Short Form Health Survey (SF-36 questionnaire), was eagerly anticipated and expected to provide strong evidence. However, MICS-CABG is still not widely distributed, which aside from the steep learning curve has been attributed to insufficient evidence from small samples with short follow-up or single-cohort series.^3^ In addition, the completeness of revascularization and patency of the angiographic graft, considered crucial controversies of MICS-CABG, have rarely been compared with conventional CABG in previous studies.

Thus, we expanded the sample size, extended the follow-up, and incorporated indices of completeness of revascularization, angiographic graft patency, and major adverse cardiac and cerebrovascular events (MACCE) in this analysis to compare MICS-CABG and sternotomy-CABG.

## Patients and Methods

### Study Population

Our institutional review board waived written informed consent for this retrospective study (IRB 00006761; December 6, 2020).

**Figure 1:**
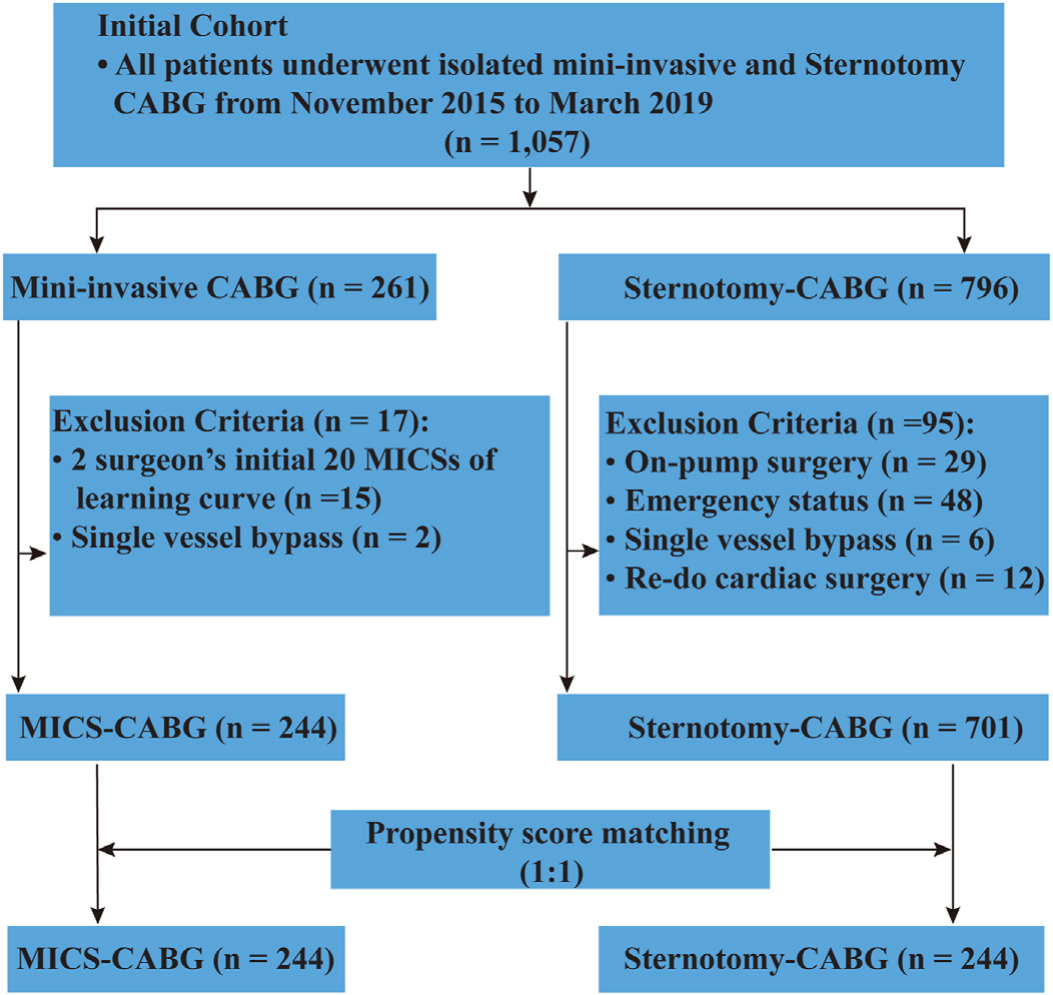
Flowchart of patient selection. (CABG, coronary artery bypass grafting; MICS, minimally invasive coronary surgery.)

Figure 1 shows the selection of patients. We retrospectively reviewed data from our institution from November 2015 to March 2019. In total, 1057 consecutive individuals who underwent first-time isolated CABG were examined. To minimize technique bias, we restricted off-pump sternotomy-CABG as the control and limited the analysis to 2 experienced surgeons (at least 20 MICS-CABG and 100 off-pump bypass operations) who have surpassed the learning curve. Exclusion criteria included emergency status, redo surgery, and single-vessel bypass. After appropriate exclusion, 945 individuals were identified, of whom 244 (25.8%) underwent MICS-CABG and 701 (74.2%) underwent sternotomy-CABG. Ultimately, 244 pairs were formed by 1-to-1 matching.

### Surgical Technique

Decision-making and procedural details for MICS-CABG are available in Supplemental Figure 1 and the Video.

### Study End Point

The primary outcomes were the completeness of revascularization (defined as a ratio of vessels deemed necessary for revascularization that were actually bypassed; ≥1 was considered complete revascularization)^5^ and the MACCE (a composite of death, myocardial infarction [MI], stroke, or repeated target vessel revascularization [TVR]). Secondary outcomes encompassed the angiography graft patency of the MICS-CABG group (the day before discharge), individual components of MACCE, and in-hospital complications. Pertinent definitions are described in the Supplemental materials.

### Statistical Methods

Continuous variables are expressed as mean (SD) or median (interquartile range); categorical variables are described as frequencies (percentages). No imputation was implemented for <1% of missing data. Continuous variables were compared by the Student *t*-test or Mann-Whitney *U* test; categorical variables used the *χ*^2^ test.

Propensity score matching was performed by the 1:1 nearest neighbor method without replacement, with a caliper of 0.2. A standardized mean difference of <0.10 was considered optimal matching. The annual surgery volume was also matched (Supplemental Figure 2). The propensity score distribution was visualized to display the postmatching covariate equilibrium (Supplemental Figures 3 and 4).

The Cox proportional hazards model was used to estimate the hazard ratios (HRs) and 95% CIs. The log-rank test was used to compare MACCE and death; the Fine-Gray test was used to compare MI, stroke, and TVR when death was considered a competing risk. All tests were 2 tailed, with a *P* value < .05 indicating statistical significance. R software (version 4.3.1; R Foundation for Statistical Computing) was used for analysis.

## Results

### Baseline Characteristics and In-Hospital Outcomes

Baseline characteristics were similar between groups (Supplemental Table 1).

Supplemental Table 2 describes the procedural characteristics and postoperative outcomes. Of 244 MICS-CABG patients, 131 (53.7%) received 2 conduits, 88 (36.1%) received 3 conduits, and 25 (10.2%) received at least 4 conduits. The completeness of revascularization was comparable between the groups (95.5% vs 96.3%; *P* = .65), and the MICS-CABG group used more arterial grafts (median [interquartile range], 1 [1-2] vs 1 [1-1]; *P* < .001) than the sternotomy-CABG group. The MICS-CABG group required fewer transfusions (11.5% vs 20.9%; *P* = .005) than the sternotomy-CABG group. The incidence of death (1.2% vs 2.0%; *P* = .73), MI (1.2% vs 1.2%; *P* > .99), reintubation (2.5% vs 0.8%; *P* = .23), and reexploration (4.5% vs 2.5%; *P* = .33) was comparable between the MICS-CABG and sternotomy-CABG groups.

Postoperative angiography was performed for 232 (95.1%) MICS-CABG patients, with 601 conduits evaluated. It revealed an overall patency of 96.2% (578/601; Supplemental Table 3).

### Follow-up Outcomes

**Figure 2:**
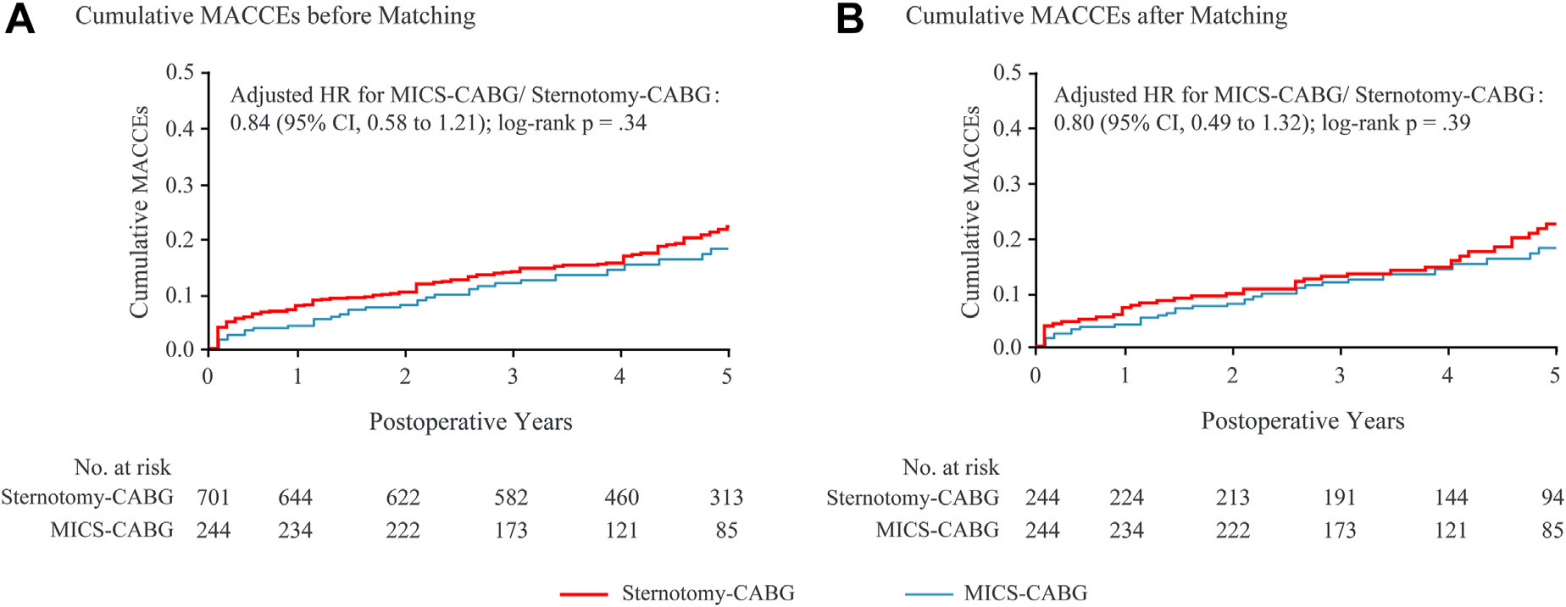
Cumulative major adverse cardiac and cerebrovascular events (MACCEs) for minimally invasive coronary surgery (MICS)–coronary artery bypass grafting (CABG) vs sternotomy-CABG (A) before and (B) after matching. (HR, hazard ratio.)

**Figure 3:**
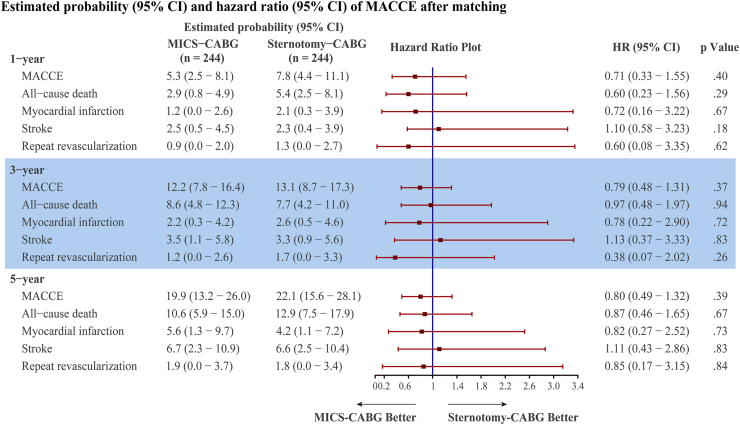
Follow-up 1-, 3-, and 5-year outcomes of minimally invasive coronary surgery (MICS)–coronary artery bypass grafting (CABG) vs sternotomy-CABG group. (HR, hazard ratio; MACCE, major adverse cardiac and cerebrovascular events.)

Throughout the mean and maximum follow-up of 48 and 60 months, the follow-up rate was 98.0% for the MICS-CABG group and 96.7% for the sternotomy-CABG group. As depicted in Figure 2, MICS-CABG was not associated with an increased 5-year MACCE (HR, 0.80; 95% CI, 0.49-1.32; log-rank *P* = .39). This association also did not differ in the Cox regression analysis (HR, 0.86; 95% CI, 0.56-1.33; *P* = .50; Supplemental Table 4). The 1-, 3-, and 5-year MACCE rates were 5.3%, 12.2%, and 19.9% for the MICS-CABG group and 7.8%, 13.1%, and 22.1% for the sternotomy-CABG group, respectively (Figure 3).

**Figure 4:**
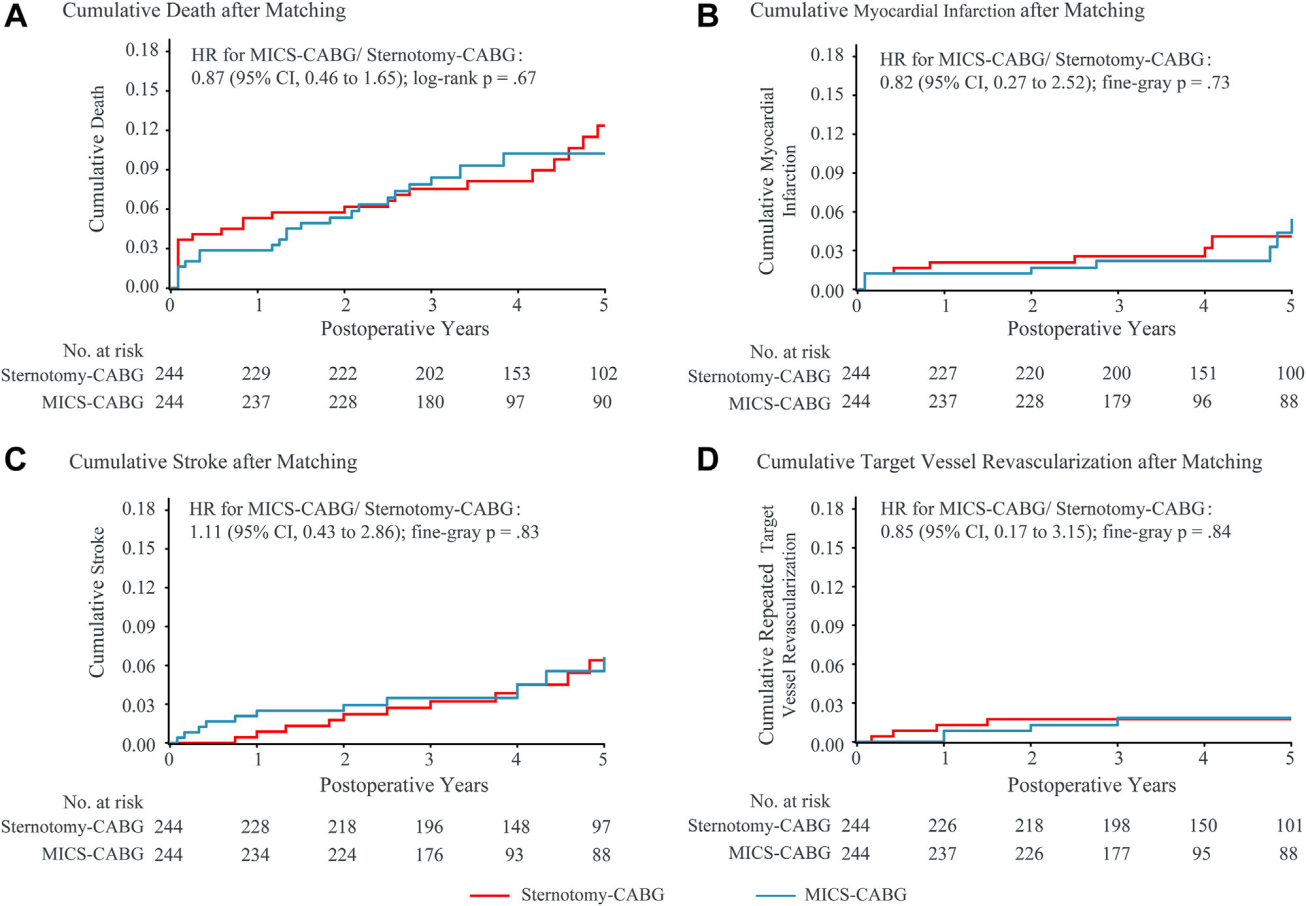
Cumulative (A) death, (B) myocardial infarction, (C) stroke, and (D) repeated target vessel revascularization for minimally invasive coronary surgery (MICS)–coronary artery bypass grafting (CABG) vs sternotomy-CABG. (HR, hazard ratio.)

As shown in Figure 4, up to 5 years, the rate of death for MICS-CABG was 10.6% vs 12.9% for sternotomy-CABG (HR, 0.87; 95% CI, 0.46-1.65; log-rank *P* = .67); the MI rate for MICS-CABG vs sternotomy-CABG was 5.6% vs 4.2% (HR, 0.82; 95% CI, 0.27-2.52; Fine-Gray *P* = .73); the stroke rate was 6.7% for MICS-CABG group vs 6.6% for the sternotomy-CABG group (HR, 1.11; 95% CI, 0.43-2.86; Fine-Gray *P* = .83); and the TVR rate for MICS-CABG was 1.9% vs 1.8% for sternotomy-CABG (HR, 0.85; 95% CI, 0.17-3.15; Fine-Gray *P* = .84).

## Comment

During the past decade, no more than 1500 cases in published series indicated that MICS resulted in fewer transfusions and expedited recovery.^6^^,^^7^ Most studies were either single-arm designs or lacked extended follow-up. This propensity score–matched study, by employing a dual-arm design and extending the follow-up to a maximum of 60 months, aimed to compare the outcomes of MICS-CABG vs sternotomy-CABG and found the following: MICS-CABG presented satisfied completeness of revascularization; the postprocedural graft patency of MICS-CABG was 96.2%; and MICS-CABG yielded noninferior 5-year MACCE to sternotomy-CABG.

Although no off-pump CABG advantages were found for long-term death or revascularization completeness,^8^ in China, the off-pump approach is still the preferred option for some experienced surgeons, whose proportion of off-pump surgery has reached more than 90%.^9^ Nonetheless, advocates of on-pump CABG raise concerns about lower rates of completeness of revascularization of MICS. In the study, the completeness of revascularization of MICS-CABG was not inferior to sternotomy-CABG (95.5% vs 96.3%; *P* = .65). The median number of grafts in the MICS-CABG group was 2 (interquartile range, 2-3), which was similar to the mean number of grafts of 2.1 (SD 0.7) and complete revascularization rate of 95% previously reported by McGinn and coworkers.^4^

Zhao and coworkers^10^ reported that 12% of the grafts had angiographic defects that had gone undetected intraoperatively. Thus, in early practice during the 2015-2019 period, we introduced postprocedural angiography as a routine strategy for quality improvement of MICS-CABG. In our study, 95.1% (232/244) of patients and 601 grafts were angiographically evaluated, with an overall graft patency rate of 96.2%. Furthermore, an additional benefit of postprocedural angiography was that any identified graft defects could be immediately addressed percutaneously. In the study, 5.3% (13/244) of MICS-CABG patients received unplanned stenting of the staged hybrid procedure.

No statistically significant difference was detected in 5-year MACCE for MICS-CABG compared with sternotomy-CABG (19.9% vs 22.1%; HR, 0.80; 95% CI, 0.49-1.32; *P* = .39), which may be attributed to the massive use of multiple arterial conduits. In the study, the MICS-CABG group received more multiple arterial conduits than the sternotomy-CABG group (26.2% vs 14.8%; *P* = .002). Three reasons were outlined: for sternotomy CABG, bilateral internal thoracic artery harvesting was associated with sternum nonunion risk, particularly for those with diabetes or osteoporosis and the elderly; for the MICS procedure, T/Y composite conduits combined with in situ bilateral internal thoracic artery were an optimal alternative to graft-to-aorta bypass; and multiple arterial conduits and T/Y composite configurations were part of the MICS “no-touch” strategy, especially for those with significant dilation or calcification of the ascending aorta.

The real-world analysis revealed that MICS-CABG demonstrated noninferior 5-year MACCE compared with sternotomy-CABG. In addition to the well-established inherent benefits of minimal invasiveness and sternum sparing, we posited that the excellent intermediate-term results of MICS-CABG vs sternotomy-CABG reside in satisfied complete revascularization and use of multiple arterial conduits.

### Limitations

There are several limitations. First, the study was conducted in a single large academic institution with the limitation of the 2015-2019 period; therefore, care must be taken in generalizing these results to a broader population. Second, despite the implementation of rigorous matching to account for baseline covariates between groups, potential unmeasured confounders can also cause treatment selection bias. Third, postoperative angiography was performed only for MICS-CABG. In addition, owing to the retrospective nature of the study, the early quality of life after surgery, which is essential, was unavailable.

### Conclusion

MICS-CABG appeared to yield similar completeness of revascularization to sternotomy-CABG and was not associated with higher intermediate-term MACCE.
