# Supplementary material for: Minimally Invasive vs Conventional Coronary Bypass Surgery for Multivessel Coronary Disease
Source: Ann Thorac Surg Short Rep. 2024 Nov 14;3(2):402–7. doi: 10.1016/j.atssr.2024.10.024 (PMC12167539; doi:10.1016/j.atssr.2024.10.024)
Supplement: Supplementary Material [file mmc2.docx]

**Supplemental material**

| **Contents** | | **Page** |
| --- | --- | --- |
| Endpoint Definitions | | 2 |
| The Decision-making of Surgical Revascularization | | 4 |
| Minimally Invasive Coronary Surgery Technique | | 5 |
| Follow-up | | 6 |
| Supplemental Figures and Tables | | 7 |
| Supplemental Figure 1 | The Decision-making of Surgical Revascularization | 7 |
| Supplemental Figure 2 | The Number of Operations Over Time Between Two Groups | 8 |
| Supplemental Figure 3 | Histogram of Propensity Score Distribution Before and After Matching | 9 |
| Supplemental Figure 4 | Standardized Mean Differences and Jitter Plot of Propensity Score Distribution Before and After Matching | 10 |
| Supplemental Table 1 | Demographic Characteristics of the MICS-CABG versus Sternotomy-CABG Groups | 11 |
| Supplemental Table 2 | Procedural Characteristics and In-hospital Outcomes of the Overall Series and Propensity Score-Matched Pairs | 14 |
| Supplemental Table 3 | Postprocedural Angiographic Outcomes of MICS-CABG | 17 |
| Supplemental Table 4 | Multivariate Cox Regression on 5-year MACCE in Overall Patients after MICS-CABG vs Sternotomy-CABG Groups | 18 |

**Endpoint Definitions**

**MACCEs** major adverse cardiac and cerebrovascular events were defined as a composite of death, myocardial infarction, stroke, or repeated revascularization.

**Death** was defined as those from any cause.

**Myocardial infarction** occurred when there were clinical signs and symptoms of ischemia that were distinct from the presenting ischemic event and met at least one of the following criteria:

1. Spontaneous (discharged from hospital after revascularization)

A. New, significant Q waves in at least two contiguous leads of an electrocardiogram;

B. Cardiac markers measured required increased CK-MB or troponin above the 99th percentile upper limit of normal and at least ≥20% above the most recent value.

1. In-hospital after CABG

The CABG-related myocardial infarction was defined by the elevation of cardiac biomarker values >10 times the 99th percentile upper reference limit in patients with normal baseline cardiac troponin values (≤99th percentile upper reference limit) plus either new pathological Q waves; new left bundle-branch block, angiographically documented new graft, or native coronary artery occlusion; or imaging evidence of new loss of viable myocardium or new regional wall motion abnormality.

**Stroke** was confirmed by a neurologist based on imaging studies and symptoms and was defined as follows:

1. A focal neurologic deficit of central origin lasting >72 hours, or
2. A focal neurologic deficit of central origin lasting >24 hours, with imaging evidence of cerebral infarction or intracerebral hemorrhage, or
3. A non-focal encephalopathy lasting >24 hours with imaging evidence of cerebral infarction or hemorrhage adequate to account for the clinical state, or
4. Transient ischemic attack is defined as an acute focal neurological deficit of vascular origin with signs and symptoms lasting less than 24 hours.
5. Retinal arterial ischemia or hemorrhage was included in the definition of stroke.

**Repeated target vessel revascularization** was defined as any repeated percutaneous coronary intervention or bypass surgery for the target vessels.

**Complete revascularization index** was defined as the percentage of deemed necessary revascularized target vessels (LAD, LCX, and RCA territory stenosis ≥ 70%, or left main trunk stenosis ≥ 50%; and diameter ≥ 1.5mm) by surgeons that were actually bypassed, which ≥ 1 was considered complete revascularization.

**Re-exploration** was defined as reoperation performed if the bleeding exceeded 200 mL/hour in the ﬁrst 3 hours or 300 mL/hour at any time, or the presence of typical hemodynamic instability or echocardiographic features of cardiac tamponade.

**Acute kidney injury** was defined as serum creatinine increase > 26.5 mmol/L or an increase to 1.5-fold from baseline within 48 hours.

**The Decision-making of Surgical Revascularization**

The decision-making is displayed in Supplemental Figure 1. The selection of MICS-CABG or sternotomy-CABG for revascularization was at the discretion of the surgeons at the clinical site, considering the patient’s clinical conditions and willingness. Angiographic criteria for CABG eligibility included the following: 1) patient with left main or bifurcation stenosis > 50%; or 2) with at least any two stenoses of >70% at the left anterior descending (LAD) territory (proximal-, mid- or distal LAD disease; diagonal or ramus intermedius vessel when large enough to warrant revascularization), left circumflex (LCX) distributions (obtuse marginal branches), and right coronary artery (RCA) territory (posterior descending artery). Clinical eligibility included angina complaints or objective examination evidence of myocardial ischemia. The following exclusion criteria were selected: 1) contraindications to MICS-CABG, such as severe left pleural adhesion and chest deformity, emergency surgery, significant cardiac dysfunctions (left ventricle end-diastolic diameter > 65 mm or left ventricle ejection fraction <40%), hemodynamic instability (need for cardiac support using an intra-aortic balloon pump, inotropic agents, or mechanical ventilation), concurrent valve or aorta surgery, left ventricular aneurysm or severe arrhythmia, hypoxemia not suitable for single-lung ventilation (partial pressure of O2 < 60 mmHg or partial pressure of CO2 > 50 mmHg), and 2) contraindications of conventional off-pump CABG, including terminal cancer, preexisting vital organ dysfunction, extensive myocardial infarction with rarely viable myocardium, insufficient vein graft conduits, and ungraftable target vessel (e.g., severely diffuse and calcification, chronic total obstructive lesion, or target vessel diameter < 1.5 mm).

**Minimally Invasive Coronary Surgery Technique**

The patient was supine with the left chest elevated 30 degrees and was anesthetized with double-lumen endotracheal intubation for single-lung ventilation. Despite the off-pump approach used in the MICS-CABG procedure, a cardiopulmonary bypass was maintained on standby. Intraoperative transesophageal echocardiography was performed to monitor cardiac performance and volume.

The procedure details are accessible through Supplemental Video. MICS-CABG was performed with direct vision through a mini-thoracotomy (a 4–6 cm incision situated between the midclavicular and anterior axillary lines of the left fourth or fifth intercostal spaces). Exposure was facilitated using the Thorac-Pro ITA tractor (Fehling Surgical Instruments Inc., Karlstein, Germany) in conjunction with the minimal intercostal retractor, with both elevated by the Rultract retractor (Rultract Inc., Cleveland, Ohio) attached to the operating table. The internal thoracic artery (ITA) was meticulously harvested with specialized surgical instruments. Before harvesting ITA, unfractionated heparin was administered to achieve an activated clotting time of ≥300 seconds. The saphenous vein graft and the radial artery were harvested and preserved for standby use. The bypass strategy was discussed with the surgeons at the clinical site. The epicardial stabilizer and apical positioner were employed to facilitate exposure of the auxiliary target territory. The distal anastomosis was performed off-pump using a 7–0 polypropylene suture, assisted by an intracoronary shunt and a water-sprayed syringe. Before heparin neutralization, the grafts were assessed using Transit Time Flow Measurement, which required a mean graft flow of ≥20 ml/min and a pulsatility index of ≤5, failing which surgical revision was mandated. The intercostal block was performed by intramuscular bupivacaine during the closure of the thorax.

**Follow-up**

As part of institutional standard practice, patients were required to undergo periodic outpatient reexaminations at 1, 6, and 12 months post-discharge and were subjected to telephone follow-up from January 2016 to November 2022. Individuals who were lost to follow-up were classified as non-events and censored.

**Supplemental Figure 1. The Decision-making for MICS-CABG**


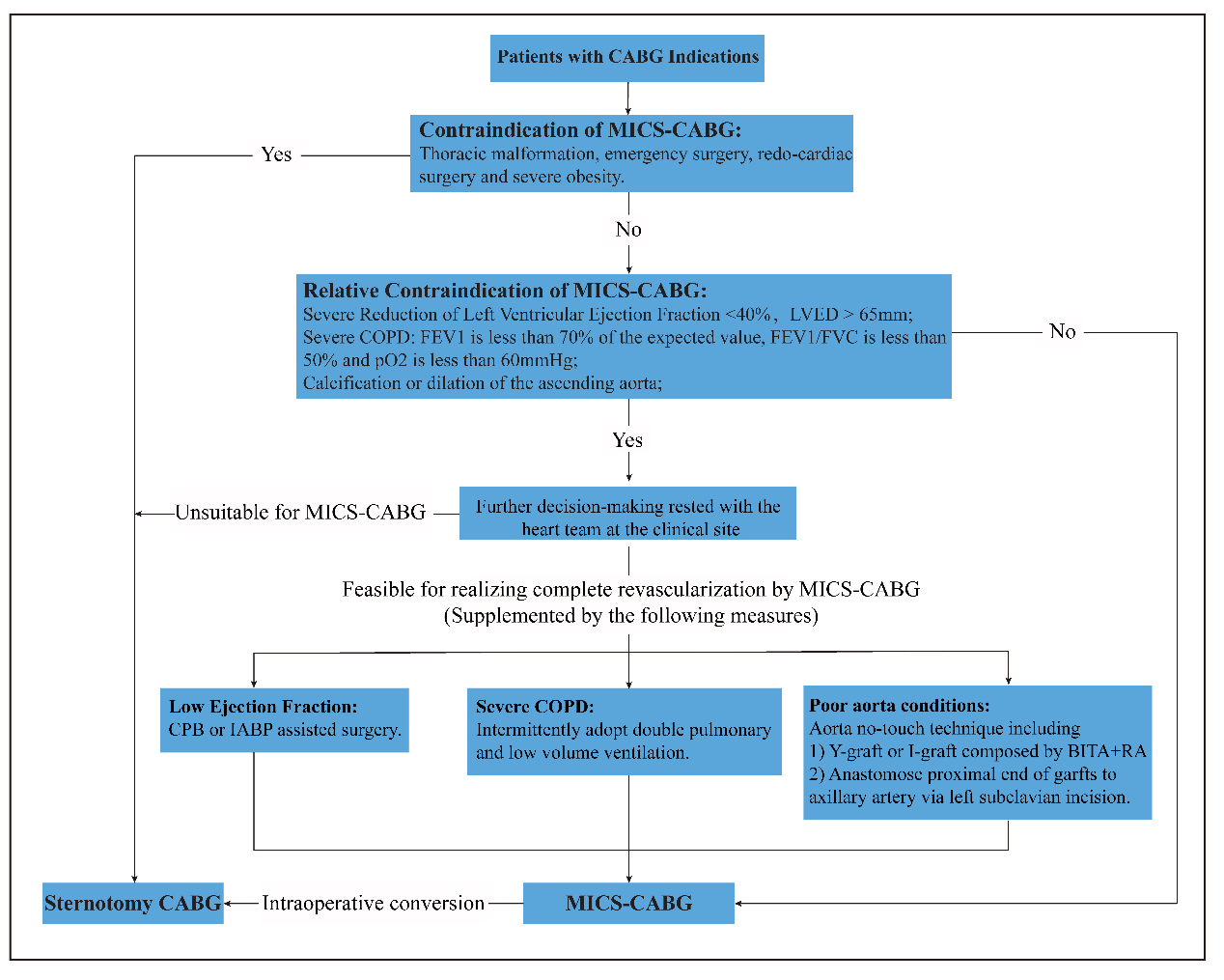


**Abbreviations:** BITA = bilateral internal thoracic artery; CABG = coronary artery bypass grafting; COPD = chronic obstructive pulmonary disease; CPB = cardiopulmonary bypass; EF = ejection fraction; FEV1/FVC = forced expiratory volume in 1 second divided by the forced vital capacity; IABP = intra-aortic balloon pump; LVED = left ventricle end-diastolic dimension; MICS = minimally invasive coronary surgery; RA = radial artery.

**Supplemental Figure 2. The Number of Operations Over Time Between Two Groups**


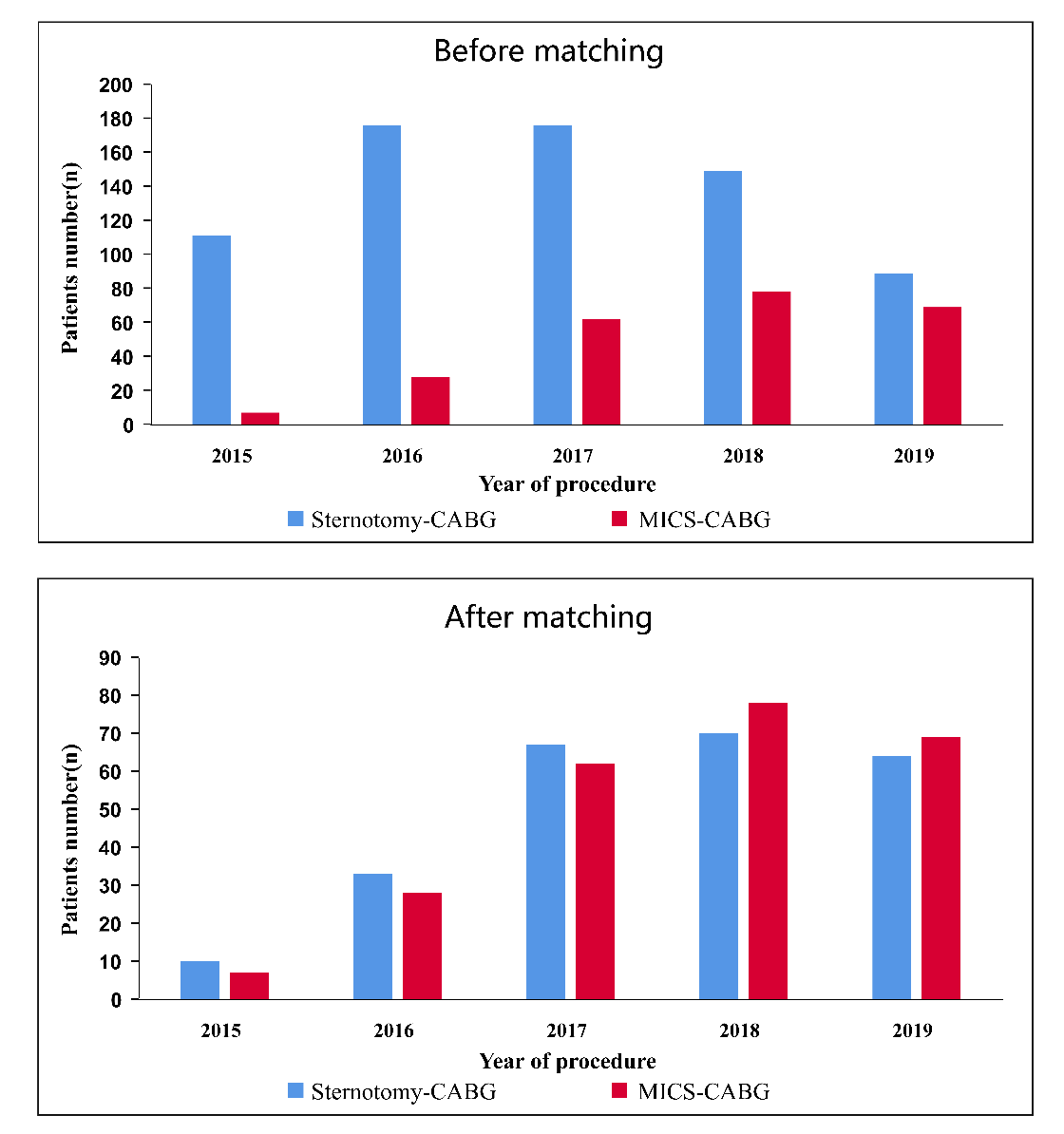


**Abbreviations:** CABG, coronary artery bypass grafting; MICS, minimally invasive coronary surgery.

**Supplemental Figure 3. Histogram of Propensity Score Distribution Before and After Matching**


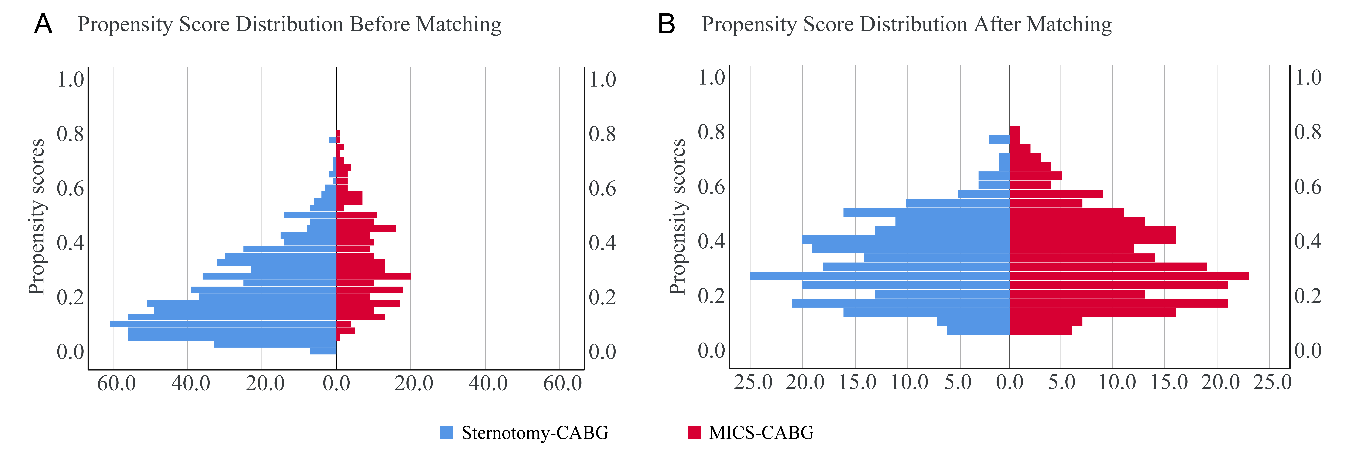


**Abbreviations:** CABG, coronary artery bypass grafting; MICS, minimally invasive coronary surgery.

**Supplemental Figure 4. Standardized Mean Differences and Jitter Plot of Propensity Score Distribution Before and After Matching**


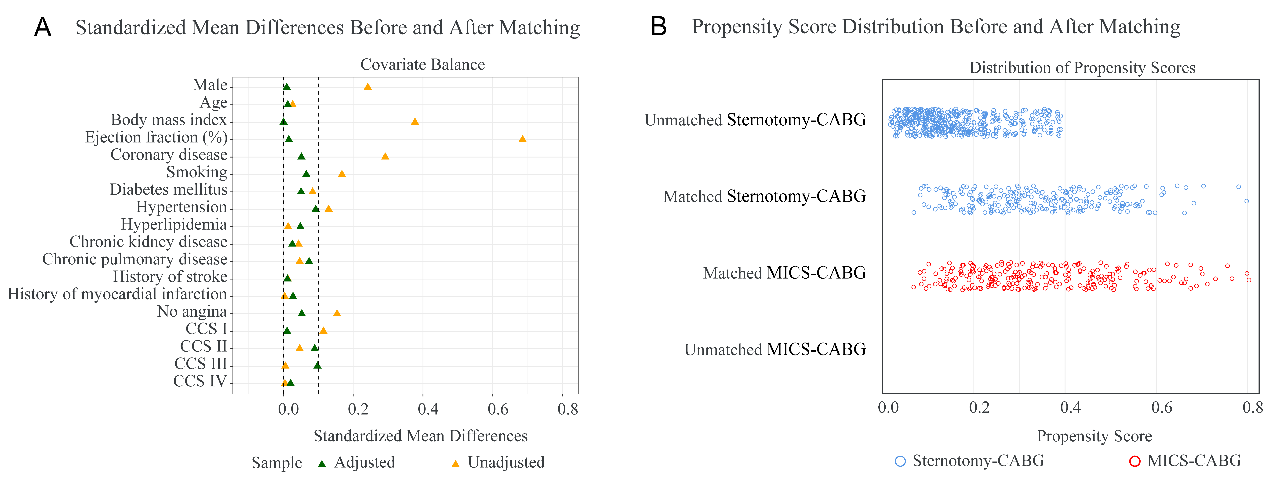


**Abbreviations:** CABG, coronary artery bypass grafting; MICS, minimally invasive coronary surgery.

**Supplemental Table 1. Demographic Characteristics of the MICS-CABG versus Sternotomy-CABG Groups**

|  | Before-PSM, No. (%) | | |  | Post-PSM, No. (%) | | |  |
| --- | --- | --- | --- | --- | --- | --- | --- | --- |
|  | MICS-CABG  (n = 244) | Sternotomy-CABG  (n = 701) | SMD, % | P value | MICS-CABG  (n = 244) | Sternotomy-CABG  (n = 244) | SMD, % | P value |
| Sociodemographic |  |  |  |  |  |  |  |  |
| Male | 197 (80.7) | 499 (71.2) | 24.2 | .004 | 197 (80.7) | 198 (81.1) | 1.0 | .91 |
| Age, mean (SD) | 62.3 (8.7) | 62.0 (9.3) | 2.7 | .72 | 62.3 (8.7) | 62.2 (9.1) | 1.3 | .89 |
| Body mass index, mean (SD) | 24.5 (3.0) | 25.7 (3.5) | 37.7 | < .001 | 24.5 (3.0) | 24.5 (3.0） | 0.1 | > .99 |
| Comorbidities |  |  |  |  |  |  |  |  |
| Smoking | 113 (46.3) | 266 (38.0) | 16.8 | .02 | 113 (46.3) | 105 (43.0) | 6.6 | .47 |
| Hypertension | 152 (62.3) | 481 (68.6) | 13.0 | .07 | 152 (62.3) | 163 (66.8) | 9.3 | .30 |
| Diabetes | 92 (37.7) | 293 (41.8) | 8.4 | .26 | 92 (37.7) | 98 (40.2) | 5.1 | .58 |
| Hyperlipidemia | 56 (23.0) | 165 (23.5) | 1.4 | .85 | 56 (23.0) | 51 (20.9) | 4.9 | .58 |
| Chronic kidney disease | 6 (2.5) | 22 (3.1) | 4.4 | .59 | 6 (2.5) | 7 (2.9) | 2.6 | .78 |
| Chronic obstructive pulmonary disease | 3 (1.2) | 5 (0.7) | 4.7 | .73 | 3 (1.2) | 1 (0.4) | 7.4 | .62 |
| Stroke | 31 (12.7) | 92 (13.1) | 1.3 | .87 | 31 (12.7) | 30 (12.3) | 1.2 | .89 |
| Myocardial infarction | 61 (25.0) | 174 (24.8) | 0.4 | .96 | 61 (25.0) | 64 (26.2) | 2.8 | .76 |
| Left ventricular ejection fraction, mean (SD) | 65.2 (9.5) | 58.7 (13.8) | 68.5 | < .001 | 65.2 (9.5) | 65.0 (10.6) | 1.6 | .87 |
| Diseased vessel |  |  | 29.2 | < .001 |  |  | 5.2 | .55 |
| Double-vessel | 46 (18.9) | 52 (7.4) |  |  | 46 (18.9) | 41(16.8) |  |  |
| Triple-vessel | 198 (81.1) | 649 (92.6) |  |  | 198 (81.1) | 203 (83.2) |  |  |
| Canadian Cardiovascular Society grading |  |  |  | .26 |  |  |  | .80 |
| I | 38 (15.6) | 80 (11.4) | 11.5 |  | 38 (15.6) | 39 (16.0) | 1.1 |  |
| II | 72 (29.5) | 222 (31.7) | 4.7 |  | 72 (29.5) | 82 (33.6) | 9.0 |  |
| III | 118 (48.4) | 337 (48.1) | 0.6 |  | 118 (48.4) | 106 (43.4) | 9.8 |  |
| IV | 10 (4.1) | 28 (4.0) | 0.5 |  | 10 (4.1) | 9 (3.7) | 2.1 |  |

Data are presented as the frequency (percentage) unless otherwise indicated. CABG, coronary artery bypass grafting; MICS, minimally invasive coronary surgery; PSM, propensity score matching; SD, standard deviation; SMD, standardized mean difference

**Supplemental Table 2.** **Procedural Characteristics and In-hospital Outcomes of the Overall Series and Propensity Score-Matched Pairs**

|  | Overall | |  | Propensity Score-Matched Pairs | |  |
| --- | --- | --- | --- | --- | --- | --- |
|  | MICS-CABG  (n = 244) | Sternotomy-CABG  (n = 701) | P Value | MICS-CABG  (n = 244) | Sternotomy-CABG  (n = 244) | P Value |
| **Procedural Characteristics** |  |  |  |  |  |  |
| Number of grafts, median (IQR) | 2 (2–3) | 3 (2–3) | < .001 | 2 (2–3) | 3 (2–3) | < .001 |
| 2 grafts | 131 (53.7) | 183 (26.1) | < .001 | 131 (53.7) | 68 (27.9) | < .001 |
| 3 grafts | 88 (36.1) | 344 (49.1) | < .001 | 88 (36.1) | 119 (48.8) | .005 |
| ≥ 4 grafts | 25 (10.2) | 174 (24.8) | < .001 | 25 (10.2) | 57 (23.4) | < .001 |
| Multi-arterial grafts | 64 (26.2) | 95 (13.6) | < .001 | 64 (26.2) | 36 (14.8) | .002 |
| Left internal mammary artery | 230 (94.3) | 636 (90.7) | .09 | 230 (94.3) | 216 (88.5) | .03 |
| Right internal mammary artery | 44 (18.0) | 4 (0.6) | < .001 | 44 (18.0) | 2 (0.8) | < .001 |
| Radial artery | 27 (11.1) | 91 (13.0) | .44 | 27 (11.1) | 35 (14.3) | .35 |
| Bilateral internal thoracic artery in situ | 13 (5.3) | 0 | < .001 | 13 (5.3) | 0 | < .001 |
| Ascending aorta “no-touch” technique | 31 (12.7) | 0 | < .001 | 31 (12.7) | 0 | < .001 |
| Complete revascularization | 233 (95.5) | NA | NA | 233 (95.5) | 235 (96.3) | .65 |
| **In-hospital Outcomes** |  |  |  |  |  |  |
| Mortality | 3 (1.2) | 24 (3.4) | .08 | 3 (1.2) | 5 (2.0) | .73 |
| Myocardial infarction | 3 (1.2) | 6 (0.9) | .89 | 3 (1.2) | 3 (1.2) | >.99 |
| Stroke | 0 | 0 | >.99 | 0 | 0 | >.99 |
| Re-exploration | 11 (4.5） | 25 (3.6) | .51 | 11 (4.5） | 6 (2.5） | .33 |
| Intra-aortic balloon pump support | 9 (3.7) | 49 (7.0) | .06 | 9 (3.7) | 6 (2.5) | .61 |
| Reintubation | 6 (2.5) | 9 (1.3) | .33 | 6 (2.5) | 2 (0.8) | .23 |
| Red blood cell transfusion | 28 (11.5) | 164 (23.4) | < .001 | 28 (11.5) | 51 (20.9) | .005 |
| Mechanical ventilation, median (IQR), h | 14 (10–18) | 15 (10–20) | .39 | 14 (10–18) | 13 (9–18) | .12 |
| Length of intensive care unit stay, median (IQR), h | 24 (18–49) | 38 (21–64) | .004 | 24 (18–49) | 25 (20–48) | .56 |

Data are presented as frequency (percentage) unless otherwise indicated. CABG, coronary artery bypass grafting; IQR, interquartile range; MICS, minimally invasive coronary surgery; NA, not applicable.

**Supplemental Table 3. Postprocedural Angiographic Outcomes of MICS-CABG**

|  | Total | Grade A | Grade B | Grades A and B | Grade O |
| --- | --- | --- | --- | --- | --- |
| Overall | 601 | 558 (92.8) | 20 (3.3) | 578 (96.2) | 23 (3.8) |
| By Conduits |  |  |  |  |  |
| Left internal mammary artery | 222 | 210 (94.6) | 9 (4.1) | 219 (98.6) | 3 (1.4) |
| Right internal mammary artery | 39 | 35 (89.7) | 2 (5.1) | 37 (94.9) | 2 (5.1) |
| Radial artery | 35 | 30 (85.2) | 5 (14.8) | 35 (100) | 0 |
| Saphenous vein graft | 305 | 283 (92.8) | 4 (1.3) | 287 (94.1) | 18 (5.9) |
| By Target Vessels |  |  |  |  |  |
| Left anterior descending artery | 228 | 217 (95.2) | 8 (3.5) | 225 (98.7) | 3 (1.3) |
| Left circumflex | 174 | 153 (87.9) | 10 (5.8) | 163 (93.7) | 11 (6.3) |
| Right coronary artery | 116 | 108 (93.1) | 0 | 108 (93.1) | 8 (6.9) |
| Diagonal artery or ramus intermedius | 83 | 80 (96.4) | 2(2.4) | 82 (98.8) | 1 (1.2) |

Data are presented as frequency (percentage). CABG, coronary artery bypass grafting; MICS, minimally invasive coronary surgery.

**Supplemental Table 4.** **Multivariate Cox Regression on 5-year MACCE in Overall Patients after MICS-CABG vs Sternotomy-CABG Groups**

| **Variables** | ***B*** | **S.E.** | **Wald** | **p value** | **Exp(*B*)** | **95.0% CI for Exp(*B*)** | |
| --- | --- | --- | --- | --- | --- | --- | --- |
|  |  |  |  |  |  | **Lower** | **Upper** |
| MICS- vs Sternotomy-CABG | -0.148 | 0.220 | 0.453 | 0.50 | 0.86 | 0.56 | 1.33 |
| Gender | 0.176 | 0.181 | 0.940 | 0.33 | 1.19 | 0.84 | 1.70 |
| Body mass index | -0.016 | 0.023 | 0.505 | 0.48 | 0.98 | 0.94 | 1.03 |
| Smoking history | 0.028 | 0.166 | 0.027 | 0.87 | 1.03 | 0.74 | 1.42 |
| Hypertension | 0.109 | 0.165 | 0.436 | 0.51 | 1.12 | 0.81 | 1.54 |
| Diabetes mellitus | 0.275 | 0.151 | 3.326 | 0.07 | 1.32 | 0.98 | 1.77 |
| Left ventricular ejection fraction | -0.019 | 0.006 | 10.969 | 0.001 | 0.98 | 0.97 | 0.99 |
| Number of grafts | -0.324 | 0.120 | 7.326 | 0.01 | 0.72 | 0.57 | 0.91 |
| Number of arterial grafts | -0.512 | 0.297 | 2.963 | 0.09 | 0.60 | 0.34 | 1.07 |
| Left internal mammary artery | -0.016 | 0.248 | 0.004 | 0.95 | 0.98 | 0.61 | 1.60 |
| Right internal mammary artery | 0.676 | 0.357 | 3.576 | 0.06 | 1.97 | 0.98 | 3.96 |
| Right coronary artery bypass | 0.206 | 0.148 | 1.933 | 0.16 | 1.22 | 0.92 | 1.64 |

**Abbreviations:** CI, confidence interval; CABG, coronary artery bypass grafting; MICS = minimally invasive coronary surgery. P Values before matching were adjusted by multivariate Cox regression, including gender, body mass index, smoking history, hypertension, diabetes mellitus, left ventricular ejection fraction, number of grafts, number of arterial grafts, left internal mammary artery, right internal mammary artery, and right coronary artery bypass.
